# Microvascular imaging of the unstained human superior colliculus using synchrotron-radiation phase-contrast microtomography

**DOI:** 10.1038/s41598-022-13282-2

**Published:** 2022-06-02

**Authors:** Ju Young Lee, Andreas F. Mack, Thomas Shiozawa, Renata Longo, Giuliana Tromba, Klaus Scheffler, Gisela E. Hagberg

**Affiliations:** 1 High Field Magnetic Resonance, Max Planck Institute for Biological Cybernetics, Tübingen, Germany; 2 Graduate Training Centre of Neuroscience, Eberhard Karl’s University of Tübingen, Tübingen, Germany; 3 Institute of Clinical Anatomy and Cell Analysis, Eberhard Karl’s University of Tübingen, Tübingen, Germany; 4 University of Trieste, Trieste, Italy; 5 Istituto Nazionale di Fisica Nucleare (INFN), Trieste, Italy; 6 Elettra – Sincrotrone Trieste S.C.p.A, Basovizza, Italy; 7 Department of Biomedical Magnetic Resonance, University Hospital Tübingen, Tübingen, Germany

**Keywords:** Neuroscience, 3-D reconstruction, X-ray tomography

## Abstract

Characterizing the microvasculature of the human brain is critical to advance understanding of brain vascular function. Most methods rely on tissue staining and microscopy in two-dimensions, which pose several challenges to visualize the three-dimensional structure of microvessels. In this study, we used an edge-based segmentation method to extract the 3D vasculature from synchrotron radiation phase-contrast microtomography (PC-μCT) of two unstained, paraffin-embedded midbrain region of the human brain stem. Vascular structures identified in PC-μCT were validated with histology of the same specimen. Using the Deriche-Canny edge detector that was sensitive to the boundary between tissue and vascular space, we could segment the vessels independent of signal variations in PC-μCT images. From the segmented volumetric vasculature, we calculated vessel diameter, vessel length and volume fraction of the vasculature in the superior colliculi. From high resolution images, we found the most frequent vessel diameter to be between 8.6–10.2 µm. Our findings are consistent with the known anatomy showing two types of vessels with distinctive morphology: peripheral collicular vessels and central collicular vessels. The proposed method opens up new possibilities for vascular research of the central nervous system using synchrotron radiation PC-μCT of unstained human tissue.

## Introduction

Microvasculature plays a fundamental role in neurophysiology, as a mean to deliver energy sources through blood vessels and remove waste metabolites through the perivascular space^1^. Dysfunction within the microvasculature is associated with cerebrovascular and neurodegenerative disease, and renders understanding of the human brain intracerebral microvasculature at a high level of detail an important topic. Comprehensive three-dimensional microvasculature imaging is essential to furthering knowledge of physiological and pathological processes within the brain and considerable efforts have therefore been made to devise effective imaging methods. However, methods developed to date have several limitations.

Several magnetic resonance imaging techniques have been developed for vessel imaging in vivo, such as susceptibility weighted imaging^2,3^ and quantitative susceptibility mapping for venography and arterial spin labeling and time-of-flight method for angiography. Langkammer et al.^4^ showed that vessels can also be detected in post mortem brain using susceptibility weighted imaging. While these methods do provide three-dimensional information about the vessel structure, they are limited in terms of spatial resolution, even when high magnetic field strengths are available^5,6^.

To enhance the visibility of the microvasculature in post mortem brain tissue, it is possible to perfuse contrast agents into the vessels. Although this is rarely practiced in human, Duvernoy perfused Indian ink through the major arteries that supply the brain. Thick slices from the Duvernoy collection were used to reconstruct the three-dimensional vasculature of one sulcus of the human neocortex using semi-automatic realignment^7^. However, the quality of the perfusion approach ex vivo is reported to be inconsistent, with factors such as post mortem time interval affecting the quality^8^. Therefore, it is unknown if branching vessels, down to the level of small-diameter capillaries can reliably be detected using this approach.

Vascular casting combined with electron microscopy has revealed rich information on cerebrovascular structure of human^9^ and macaque monkey^10^. Similarly, microtomography is also useful for performing complete vascular imaging of the brain for small animals such as rats and mice^11,12^. These methods are suitable for animal models, because the post mortem interval time can be minimized and the contrast agent can be perfused before vessels collapse. However, with human samples, several hours of post mortem interval time is unavoidable and perfusing solution into the vessels becomes challenging.

Synchrotron-radiation phase-contrast microtomography (PC-μCT) has been successfully used for vascular imaging in animal models perfused with contrast agents^13–17^. Preliminary results suggest that some vessel detail is also possible to discern in mouse spinal cord after perfusion even in absence of contrast agents^18^. For human brain tissue, many groups prefer to use stains for increased tissue contrast^19–21^. Only a few studies describe PC-μCT results for vascular imaging of unstained human brain^22,23^. In the present work, we explored the possibility to use PC-μCT to reveal microvasculature in unstained human brain stem samples. We developed an automated workflow to segment the vessels based on edge information at the boundary between the vascular space and neuronal tissue. We show that it can be used to identify vessels using PC-μCT data obtained at two image resolutions. From the high-resolution images, we obtained values for the vessel volume fraction, vessel length and apparent diameter which were in agreement with previous studies of human tissue. The presented method thus offers new potential for investigating microvasculature in unstained human brain tissues using PC-μCT.

## Results

### Edge-based segmentation for vessel detection

**Figure 1:**
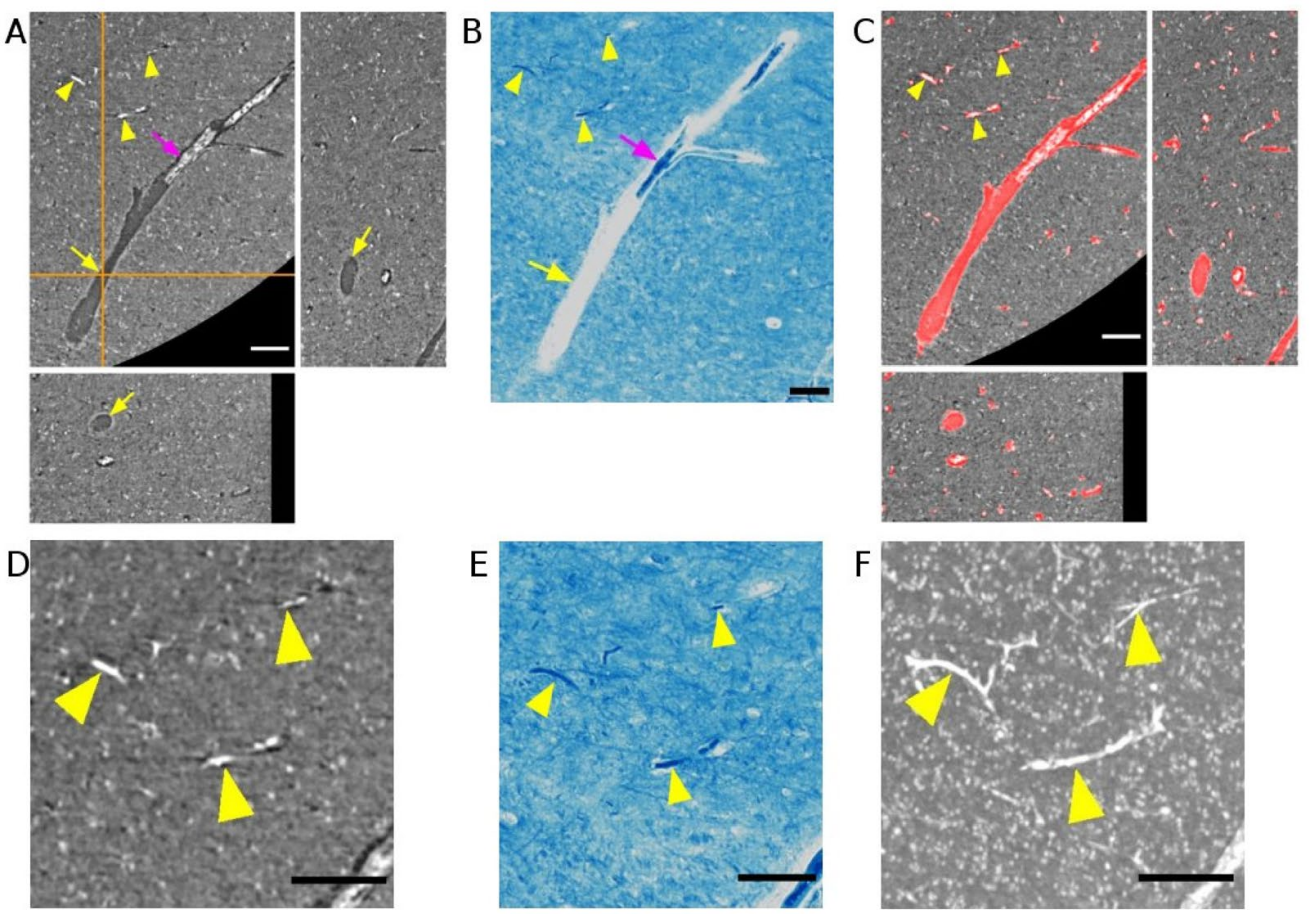
Validation of vessels found in phase-contrast microtomography by comparing with histology. (**A**) Orthogonal views of a blood vessel in a 0.94 µm voxel size microtomography image. The orange cross-hair indicates the location of the selected orthogonal planes. The vessel is characterized both by hyperintense parts with collapsed blood vessel (magenta arrow) and hypointense parts with voids (yellow arrow). (**B**) Microscope image of an approximately matching region of the same sample. The section is 10 µm thick and was stained with the Klüver-Barrera technique. (**C**) Binary vessel mask (red) obtained with the proposed segmentation method is overlayed on the image shown in A. Note that capillaries are well segmented (yellow arrowheads). (**D**) Zoomed inset of A. (**E**) Zoomed inset of B. (**F**) Maximum intensity projection of the inset region shown in D across 28 µm depth, showing an extended trajectory of the capillaries. Scale bars = 100 µm.

Vascular structure could be identified in PC-μCT (Fig. 1A) and was verified with microscopy images of matching stained histological sections of the same specimen (Fig. 1B, Supplementary Fig. S1). Within the vascular space, there were remains from collapsed blood vessels, with and without the presence of red blood cells (Supplementary Fig. S1). But in other parts, the vascular space was found empty. The orthogonal view of the PC-μCT in Fig. 1A shows that the lower part of the vascular space is indeed void. Such void vascular spaces were found in other regions as well. We suspect that these alterations occurred during the tissue preparation process. The inconsistent contrast within vascular spaces called for a segmentation method that can detect vessels, regardless of vessel appearance. By using an edge-based method, we extracted vascular structures from the boundaries between the vascular space and the tissue. Note that the output (Fig. 1C) successfully displays the whole region of the vascular space, including the parts with and without blood vessel remains.

In the two-dimensional plane, it is difficult to judge whether small structures (arrowheads in Fig. 1C) are part of the vasculature. Insets of PC-μCT (Fig. 1D) and microscope image (Fig. 1E) show that these fragments can be classified as vessels. Maximum intensity projection of PC-μCT across depth (Fig. 1F) further demonstrates that these tubular structures have extended trajectory in accordance with vascular structure.

To validate our segmentation method, we randomly selected 100 voxels within each vascular map and performed a visual control (Supplementary Fig. S2). The false positive rate was 5.6 ± 1.8%. The CD34 stain images provided additional evidence that the vessel segmentation worked reliably (Supplementary Fig. S3).

### Segmentation results from PC-μCT with 0.94 µm isotropic voxel sizes

**Figure 2:**
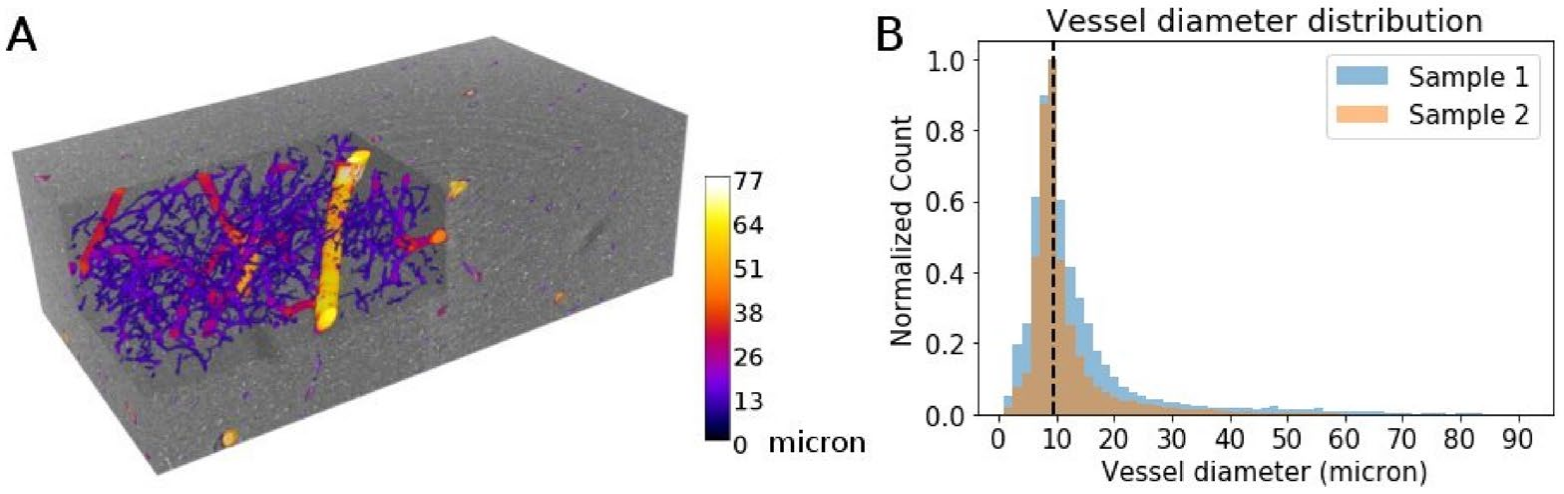
Microvasculature segmented from 0.94 µm voxel size phase-contrast microtomography. (**A**) The segmentation result from one of the regions of interest from sample 2. The vasculature is color-coded in terms of vessel diameter. The unit of the color bar is µm. The width, height and depth of the stack is 2.09 mm, 1.14 mm and 0.66 mm, respectively. (**B**) Vessel diameter distribution from the two samples. The black dashed line indicates the center of the most frequent bin.

As shown in Fig. 2A and in supplementary video, vessels with different size of diameters are successfully extracted. Similar to prior work on post mortem human cortex^21,24^, the vessel diameter distribution shows a long tail towards larger vessels (Fig. 2B). The peak of the vessel diameter is between 8.6–10.2 µm, which is in accordance with a recent study that used absorption-contrast microtomography on silver impregnated human brain^21^.

The total vessel volume fraction ranged between 2.4–4.4% and the vessel length density ranged between 269.60–449.54 mm/mm^3^ (Table 1). These parameters are within the range of reported values from previous studies of the human brain^7,25,26^. Collectively, we show that the proposed method can effectively extract microvasculature.

**Table 1:** Summary of vascular features from 0.94 µm voxel size phase-contrast microtomography.

	Vessel volume fraction (%)	Vessel length density (mm/mm^3^)
	Mean + SD	Mean + SD
Sample 1	4.0 ± 0.3	376 ± 53
Sample 2	2.8 ± 0.3	332 ± 52

For each sample, the mean and standard deviation (SD) across regions-of-interest (cfr Fig. S4) are shown.

### Segmentation results from PC-μCT with 4.94 µm isotropic voxel sizes

**Figure 3:**
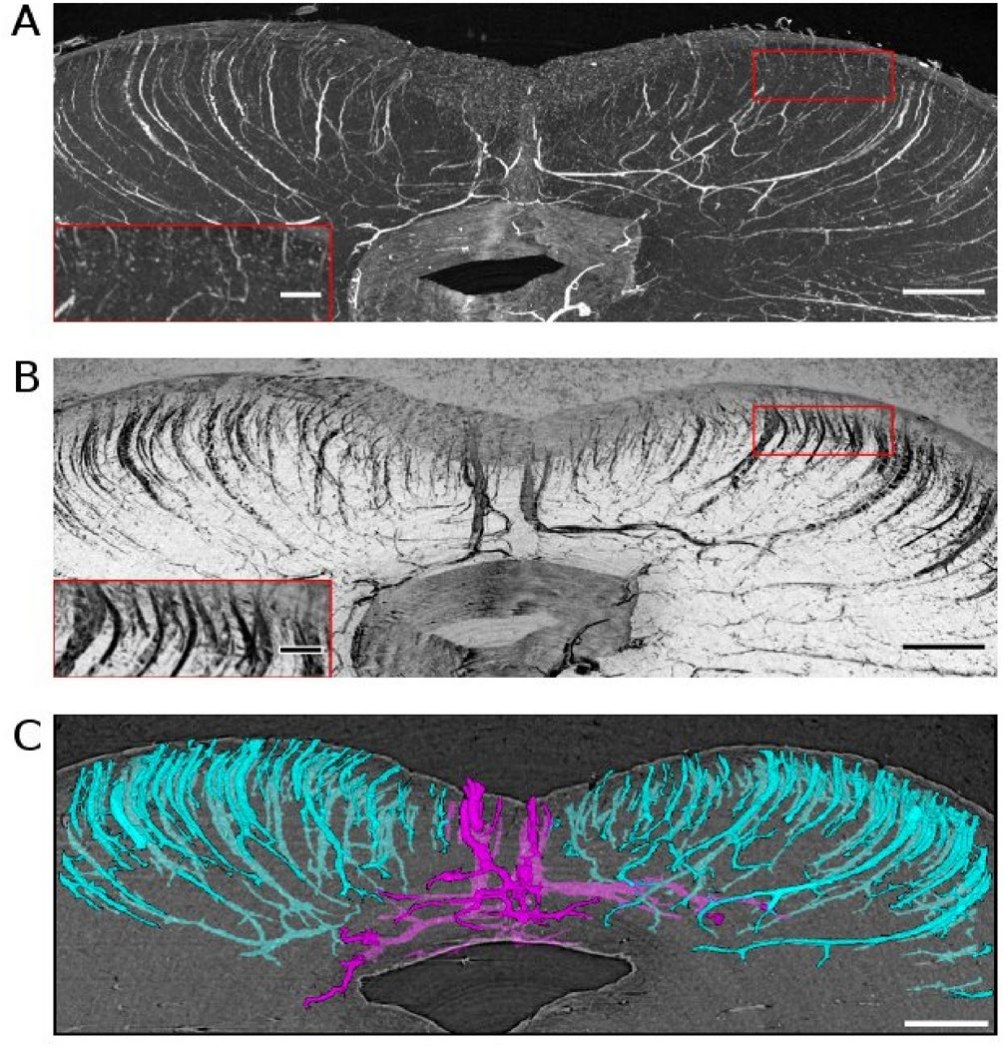
Vasculature from 4.94 µm voxel size phase-contrast microtomography. (**A**) Maximum intensity projection of sample 1 with zoomed inset showing the appearance close to the tissue surface. (**B**) Minimum intensity projection of sample 1 with zoomed inset from the same region as in A. Note the improved conspicuity of the vessels close to the tissue surface. (**C**) Three-dimensional view of the collicular vessels after segmentation. Peripheral collicular vessels are shown in cyan and central collicular vessel are shown in magenta. Scale bars = 1 mm. Scale bars of insets = 0.5 mm.

Similar vessel conspicuity was observed in the PC-μCT images acquired at a coarser resolution, which allowed a larger coverage of the specimen within similar measurement times. The difference between maximum (Fig. 3A) and minimum intensity projections (Fig. 3B) shows again the inhomogenous contrast within the vascular space. In addition, we observed that close to the pial surface, blood vessel remains were often lost, resulting in hypointensity. Deeper inside the tissue, such remains were still present resulting in hyperintensity. By using the edge-based segmentation approach, we were able to extract vascular structures independent of the presence of blood vessel remains.

Two types of vessels could be identified in the superior colliculi based on their entrance point into the brain stem: central collicular vessels (magenta in Fig. 3C) that penetrate from the intercollicular sulcus and peripheral collicular vessels (cyan in Fig. 3C) that penetrate from the surface of the superior colliculus. The two types showed distinct morphology as also described in Duvernoy^8^. Central collicular vessels penetrated the tissue along the medial axis, then made an abrupt turn ramifying laterally. Peripheral collicular vessels followed concave trajectories from the surface of the superior colliculus towards the periaqueductal gray.

Using the coarser sampling, the relative contribution of vessels with small diameters diminished. Most vessels detected had a diameter of 46.1 µm, suggesting that different ensembles of vessels can be captured depending on the voxel size. In summary, the well-known vessel of the posterior midbrain, composed of the central and peripheral collicular vessels, could be mapped using 4.94 µm voxel size images, allowing investigations of vascular morphology from an extended region of interest at the cost of more detailed information regarding the capillary network.

### Length estimates of short and long peripheral collicular vessels

In order to further characterize the peripheral vessels detected within the superior colliculus with a voxel size of 4.94 µm, we used the 0.94 µm voxel size PC-μCT data after downsampling to 1.88 µm. Working with the downsampled images allowed to remove the capillary network from the segmentation, while leaving larger vessels intact.

**Figure 4:**
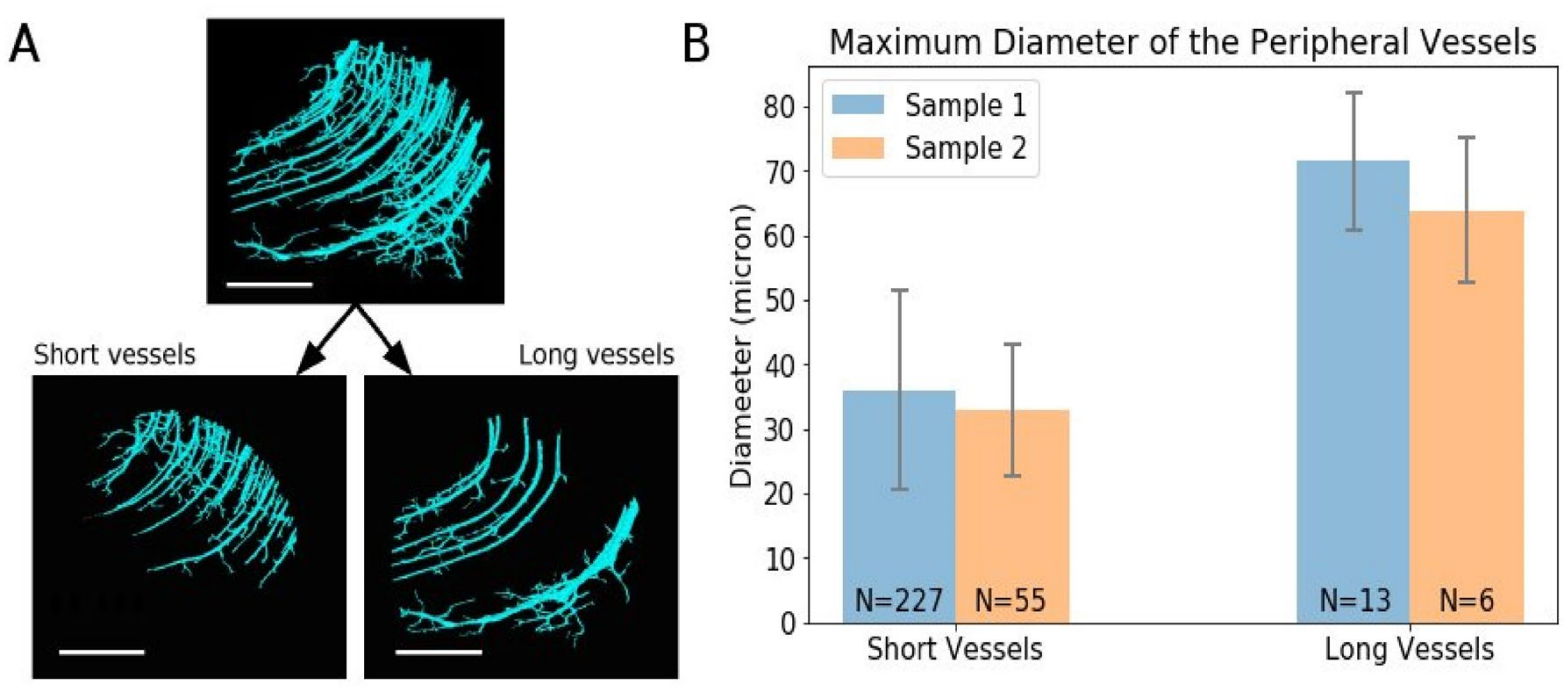
Identifying short and long peripheral collicular vessel. (**A**) Peripheral collicular vessels from sample 2. Top panel shows all the peripheral vessels. Bottom left panel shows the short peripheral vessels that end within the field of view. Bottom right panel shows the long peripheral vessels that reach the other end of the field of view. Scale bars = 1 mm. (**B**) The bar graph shows the average maximum diameter of the peripheral collicular vessels. The error bars indicate the standard deviation. N indicates the number of vessels.

In this data, we could confirm that the peripheral collicular vessels penetrate through concave trajectories (Fig. 4A). In accordance with the description in Duvernoy^8^, we could distinguish between short vessels that penetrate into the middle zone of the superior colliculus and long vessels that traverse the superior colliculus completely and end at the periacqueductal gray. The vessels that ended within the field of view were categorized as short vessels; and the vessels that reached the edge of the field of view were categorized as long vessels. The maximum diameter of the two vessel types were found to be significantly different (Student’s *T*-test *p*-value < 1.0 * 10^−19^) (Fig. 4B). The maximum diameter of the long vessels was about twofold that of the short vessels.

## Discussion

In this study, we demonstrate a workflow for three-dimensional microvascular imaging using PC-μCT of the unstained human brain stem. We developed a segmentation pipeline which does not only depend on the presence of hyperintense signals from the blood vessels, but rather detect the boundaries between tissue and vascular space by using an edge-based detection method. Our method successfully extracted vessels from PC-μCT images obtained with different voxel sizes. We validated this vessel structure by comparing with microscope images from the same samples. With 0.94 µm voxel size PC-μCT images, we traced the microvasculature and found the capillary diameters to be around 9 µm. From 4.94 µm voxel size PC-μCT images, we revealed the three-dimensional vasculature of the posterior midbrain and identified different types of collicular vessels with distinctive trajectories.

Imaging unstained soft tissue with X-rays poses challenges. Soft tissues have low X-ray absorption, resulting in poor visibility when imaged using absorption-based tomography. With coherent X-ray beams from synchrotron light sources, we were able to obtain enhanced image quality by exploiting the propagation-based phase-contrast method. The phase-contrast method uses not only absorption but also phase information, providing improved contrast-to-noise ratio to distinguish between different biological compartments^19,27^.

Prior works have provided evidence that PC-μCT of unstained human brain contains information about vascular structure. Hieber et al.^28^ showed that a Frangi vesselness filter, which is sensitive to tubular structures, can identify some vessels from the unstained human cerebellum. Töpperwien et al.^22^ used a region growing method, which required manually selected seed regions, to segment the vasculature from the unstained human hippocampus. The focus of these studies were not on vascular imaging, leaving room for improvements. Most recently, Miocchi et al.^18^ has proposed a segmentation method using three-dimensional Gaussian steerable filters for vessel enhancement and has demonstrated its effectiveness in unstained mouse spinal cord. However, this method depended on a consistent contrast from the vascular structure and was not suitable for our data.

In the obtained PC-μCT images from unstained human brain stems, we observed that vessels have inhomogeneous contrast. Vascular spaces exhibited hyperintensity where hemoglobin-containing red-blood cells and collapsed vessel walls were present and hypointensity when they were absent. Our edge-based method can be used to segment the vascular structure regardless of such variable intensity profiles within the vascular space. We foresee that the proposed method will also be useful for vascular imaging of post mortem brain samples from biobanks, where blood vessel remains may be lost due to prolonged storage in fixative solution.

There are only a small number of studies on the capillary structure of human brain. Earlier studies that injected contrast agent into the vasculature suggested the capillary diameter to be about 7–7.5 µm^9^ or 6.47 µm^7^. More recently, a synchrotron radiation imaging of a Golgi impregnated human neocortex found the capillary diameter to be 7–9 µm^21^, consistent with our results of unstained superior colliculus where the most frequently observed vessel diameter was between 8.6–10.2 µm. The slight difference in capillary diameter can depend on whether the capillary wall has been included or not. In the studies of Reina-De La Torre et al.^9^ and Lauwers et al.^7^ a contrast agent was used as the source for vascular information and their results likely represent the inner diameter of the capillaries. On the other hand, the results from Saiga et al.^21^ and our study probably include the capillary walls resulting in the outer diameter of the capillaries.

Vessel length density is usually computed by the total vessel length divided by the total vessel volume. This measure varies drastically depending on whether changes in the tissue volume during the experiment, such as tissue shrinkage, is taken into account or not. Thus, it is difficult to compare vessel length density between studies, as the shrinkage ratio is not always reported. A couple of studies on human neocortex reported the vessel length density to be between 400– 500 mm/mm^3^^7,25^. In another study on human superior colliculus, vessel length density showed a decrease with ageing, ranging from 300 mm/mm^3^ at age 34 to140 mm/mm^3^ at age 82^26^. These studies used different tissue processing methods and did not report the shrinkage ratio. Vessel length density from our data (subjects aged 74 and 81 years) ranged between 188–288 mm/mm^3^ without shrinkage correction and 294–450 mm/mm^3^ with shrinkage correction. Although it is difficult to directly compare our result with prior studies, both shrinkage corrected and uncorrected vessel length density are within the range of previously reported measures.

With the approach performed in this study, it was possible to visualize the highly structured arrangement of the vessels in the human midbrain. Particularly, the peripheral collicular vessels presented highly structured trajectories, penetrating across the layers of the superior colliculus in an orderly fashion. Using higher spatial resolution of the PC-μCT acquisition, it was also possible to segment the capillary network. To our knowledge, this is the first time that the three-dimensional vasculature of the human superior colliculi is reported. With more acquisitions, one could obtain the complete three-dimensional vasculature of the entire human brain stem. In future studies, we want to further develop the method to categorize vessels as arteries or veins. In summary, this method opens up new possibilities for vascular imaging of post mortem human brain samples.

## Methods

### Sample preparation

Human brain stem samples (N = 2) were collected at the Institute of Clinical Anatomy and Cell Analysis, Department of Anatomy, Eberhard Karls University of Tübingen (see Supplementary Table S1 for more detail). The body donors gave their informed consent in concert with the declaration of Helsinki for research purposes. The procedure was approved by the ethics commission at the Medical Department of the University of Tübingen. The donors had no history of neurological disease. The samples were fixed in formaldehyde solution (Roti®-Histofix 4% phosphate-buffered formaldehyde solution, pH 7 from Carl Roth GmbH + Co. KG, Karlsruhe, Germany; 140 mM NaCl and 2.7 mM KCl from Sigma-Aldrich Chemie GmbH, Merck KG, Taufkirchen, Germany) for a minimum of 4 weeks. The brain stems were cut into 1 cm thick slices perpendicular to the brain stem axis. The slices were embedded in paraffin. Paraffin blocks that included superior colliculus were selected for PC-μCT measurements.

### Image acquisition and reconstruction

The measurement took place at SYRMEP (SYnchrotron Radiation for MEdical Physics) beamline from Elettra Synchrotron Facility (Trieste, Italy) using propagation-based phase-contrast method. The white beam setup with a 1 mm Silicon filter was used, resulting in a mean beam energy of 20.7 keV (mode = 19.6 keV) and energy flux of 1.1 × 10^11^ photon/s/mm^2^. Each image was acquired with 3600 projections around 360° using half-acquisition mode^29^ by a water-cooled 16-bits CMOS camera (Hamamatsu C11440-22C-Flash4.0 v2). Following calculations from^30^, the samples were measured at two different detector-to-sample-distances (DSDs); 200 mm for 0.94 µm isotropic images and 900 mm for 4.94 µm isotropic images. The exposure time was 200 ms for each projection.

We used the SYRMEP Tomo Project software (version = 1.3.2) to reconstruct the images^31^. First, the sinograms were stitched using automatic center-of-rotation estimation^32^ sometimes assisted by manual correction. Then, we applied flat-field correction followed by ringing artefact removal^33^. The single-distance phase-retrieval algorithm developed by Paganin et al.^34^ was used which requires a value for the ratio of the real part (delta) over the imaginary part (beta) of the complex refractive index *n* = 1 − *δ* + *iβ*. The delta over beta values applied were 20 in DSD 200 mm projections and 50 in DSD 900 mm projections. Filtered back projection was applied for image reconstruction.

Finally, the paraffin embedded samples were serially sectioned with a microtome at 10 µm. Selected sections were stained with the standard Klüver-Barrera staining protocol, or with rabbit anti-CD34 antibodies (Abcam ab81289) and detected with goat-anti-rabbit secondary antibodies, conjugated to Alexa Fluor 546 (Molecular Probes/Thermofisher). Klüver-Barrera stained slides were digitized using a Zeiss Axio scan microscope (Zeiss, Jena, Germany). Immunofluorescent CD34 stained slides were imaged using the tile function of a Zeiss Axio Imager.Z1 fluorescence microscope (Zeiss, Jena, Germany).

### Vessel segmentation

Segmentation of the vessels was performed in FIJI^35,36^ using 3D ImageJ Suite^37^ and MorpholibJ^38^ plugins.

**Figure 5:**
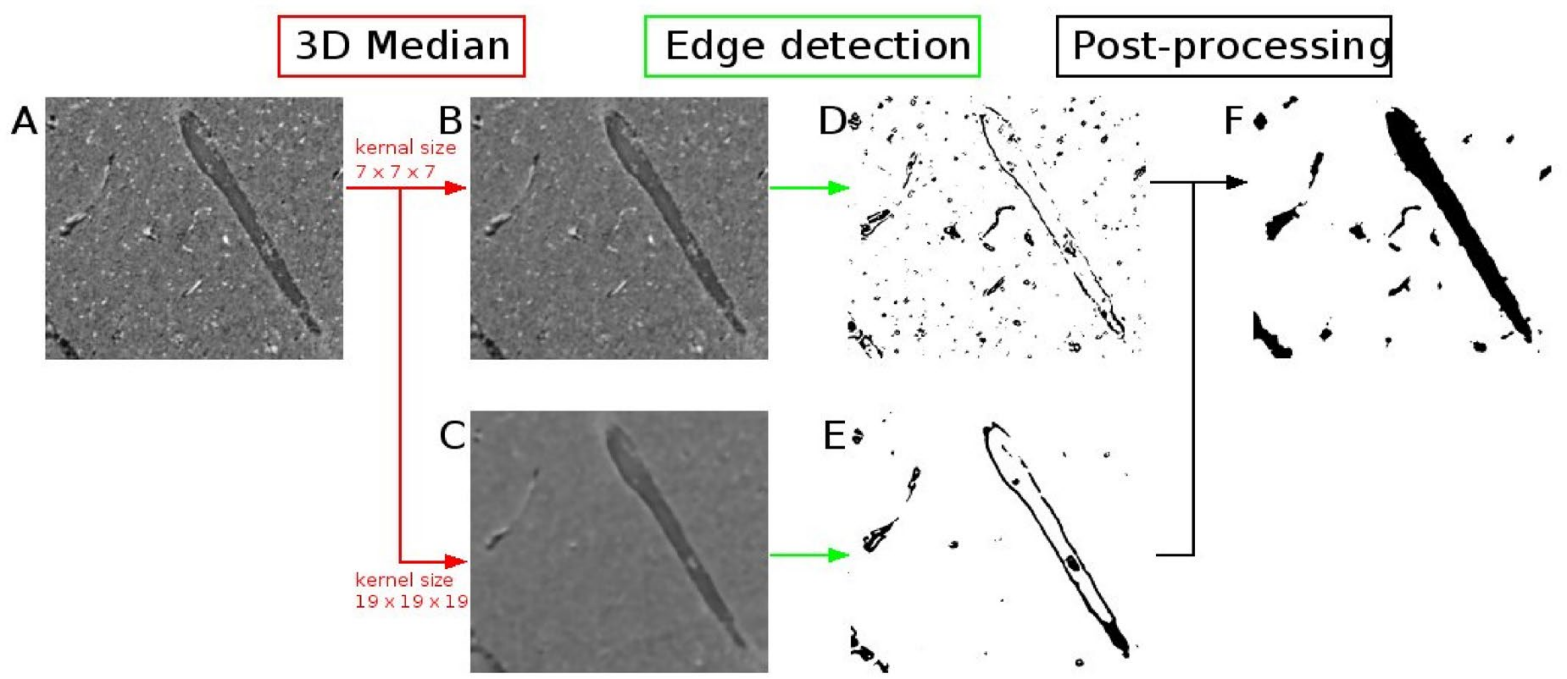
Vessel segmentation from phase-contrast microtomography with 0.94 µm isotropic voxel. (**A**) Phase-contrast microtomography from sample 1, containing several vessels (image height = 0.5 mm, width = 0.6 mm). Intermediate steps show results of applying median filter (**B**, **C**) and Deriche-Canny edge detection (**D**, **E**). Post-processing includes island removal, closing operation and fill holes. (**F**) Segmentation result shown as a binary image.

First, we aimed to extract the microvasculature from 0.94 µm voxel size PC-μCT images. We selected region-of-interests within the superior colliculus (Supplementary Fig. S4). Figure 5A–F shows the segmentation process. In order to segment vessels of all sizes, median filters with two different kernel sizes were used followed by Deriche-Canny edge detection. Median filter with a 7 × 7 × 7 kernel size allowed for preservation of capillary structures (Fig. 5B,D) and a 19 × 19 × 19 kernel size allowed for preservation of larger vascular structures with smaller edge contrast (Fig. 5C,E). Then the edge information from two filtered images (Fig. 5D,E) was combined by a logical OR operation. Lastly, three additional post-processing steps were performed: small objects with volumes smaller than 2.5*10^3^ µm^3^ were considered noise and were removed, three-dimensional closing was used for connecting the vessel structure, and the fill holes operation was performed to remove cavities within the vascular spaces (Fig. 5F and Supplementary Table S2).

**Figure 6:**
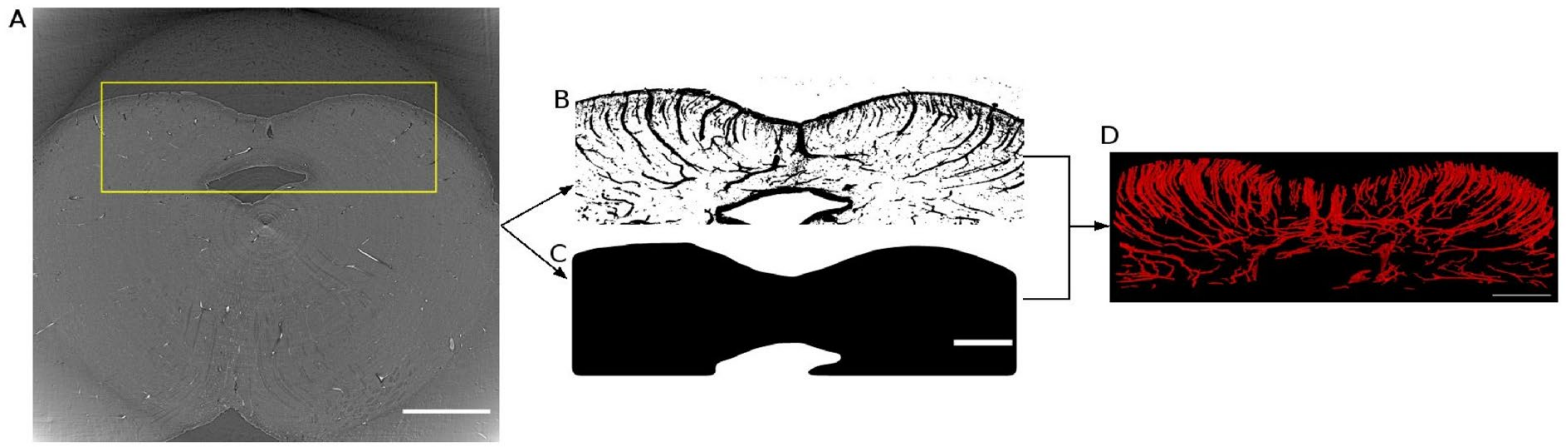
Vessel segmentation from phase-contrast microtomography with 4.94 µm voxel size. (**A**) Phase-contrast microtomography from sample 1. The yellow rectangle indicates the selected region covering the posterior midbrain. (Scale bar = 3 mm) (**B**) Result of applying Deriche-Canny edge detection on the selected region. Here, we present a maximum intensity projection of the edge detection result over 0.3 mm in Z direction for better visualization. (**C**) Mask used to identify the tissue (Scale bar = 1.5 mm). (**D**) Vessel segmentation result from the region with a 1.5 mm depth along the rostro-caudal direction.

Next, we used 4.94 µm voxel size images to segment the gross vasculature from the posterior part of the midbrain including the superior colliculi (Fig. 6A). For this analysis, median filtering was not required and the Deriche-Canny edge filter was directly applied (Fig. 6B). Since the selected region included some paraffin, the boundaries between paraffin and the tissue were also detected by edge filtering. An additional step was added in order to exclude these boundaries. We obtained the mask of the tissue (Fig. 6C) by using the region-growth segmentation function in ITK-SNAP^39^ and used it to mask out the irrelevant edges. Then the post-processing steps, including island removal, closing, and fill holes, were applied to get the final result (Fig. 6D and Supplementary Table S2).

Lastly, the entire field-of-view covered by the 0.94 µm voxel size images were evaluated (Supplementary Fig. S4). After downscaling using a 0.5 ratio and trilinear interpolation, we yielded 1.88 µm voxel size images. Then the vasculature was segmented using a 7 × 7 × 7 median filter followed by Deriche-Canny edge detector. The post-processing steps consisted of applying a circular mask to remove the edges at the border of the circular field-of-view, removing isolated small objects, applying the closing and fill hole operation (Supplementary Table S2). After vessel segmentation from each image, we stitched the images from the same samples using a phase-correlation based stitching method^40^.

### Vessel analysis

Vascular density was calculated from 0.94 µm voxel size images in terms of vessel volume fraction and vessel length density. Vessel volume fraction was computed as: [Vessel volume]/[Total volume] * 100. With Skeletonize3D plugin and Analyze Skeleton plugin implemented in Fiji, centerlines of the vessels were extracted using a three-dimensional thinning algorithm^41^, followed by pruning branches shorter than 25 µm. Vessel length density was computed as : [Total length of skeleton]/([Total volume] * [Shrinkage factor]^2^). Tissue shrinkage factor was estimated by comparing the total volume of sample 2 before and after paraffin embedding (Supplementary Fig. S5). Vessel diameter was calculated by extracting local thickness from each point of the skeleton using Local Thickness plugin^42^.

For the 4.94 µm voxel size images, the central and peripheral collicular vessels were identified through geodesic reconstruction using MorpholibJ plugin^38^. Intercollicular sulcus was used as a seed region for three-dimensional reconstruction of the central collicular vessels. Likewise, the peripheral collicular vessels were selected by using the surface of the superior colliculus as seed region.

For the 1.88 µm voxel size images, which were downsampled from the originally 0.94 µm voxel size images, the peripheral collicular vessels were also selected using geodesic reconstruction. The vessels were then divided into two groups based on length: short vessels that ended within the field-of-view and long vessels that reached the end of the field-of-view. The maximum diameter of these vessels were calculated based on the largest inscribed sphere using MorpholibJ plugin^38^.

## Supplementary Information

Supplementary Information 1.

Supplementary Video 1.

## Data Availability

Algorithms used in the suggested segmentation method are all open-source. Repositories are included in the reference section. Supplementary material and video include multi-modal data analyzed in this paper.
