# Supplementary material for: Microvascular imaging of the unstained human superior colliculus using synchrotron-radiation phase-contrast microtomography
Source: Sci Rep. 2022 Jun 2;12:9238. doi: 10.1038/s41598-022-13282-2 (PMC9163179; doi:10.1038/s41598-022-13282-2)
Supplement: Supplementary file 1 — Supplementary Information 1. [file 41598_2022_13282_MOESM1_ESM.pdf]

Microvascular imaging of the unstained human superior colliculus using synchrotron-radiation phase-contrast microtomography

Ju Young Lee[1,2]*, *Andreas F. Mack*[3], *Thomas Shiozawa*[3], *Renata Longo*[4,5], *Giuliana Tromba*[6], *Klaus Scheffler*[1,7] *and Gisela E. Hagberg*[1,7]

[1] High Field Magnetic Resonance, Max Planck Institute for Biological Cybernetics, Tübingen, Germany
[2] Graduate Training Centre of Neuroscience, Eberhard Karl's University of Tübingen, Tübingen, Germany
[3] Institute of Clinical Anatomy and Cell Analysis, Eberhard Karl's University of Tübingen, Tübingen, Germany
[4] University of Trieste, Trieste, Italy
[5] Istituto Nazionale di Fisica Nucleare (INFN), Trieste, Italy
[6] Elettra – Sincrotrone Trieste S.C.p.A, Basovizza, Italy
[7] Department of Biomedical Magnetic Resonance, University Hospital Tübingen, Tübingen, Germany

Corresponding author details:

Ju Young Lee

High Field Magnetic Resonance Max Planck Institute for Biological Cybernetics, Tübingen, Germany

Graduate Training Centre of Neuroscience, Eberhard Karl's University of Tübingen, Tübingen, Germany

Address: Max Planck Institute for Biological Cybernetics, Room 3.B.02, Max-Planck-Ring 11, 72076, Tübingen, Germany.

Email: ju.young.lee@tuebingen.mpg.de

Table S1. Sample information

	Sample 1	Sample 2
Age (years)	74	81
Sex	female	female
Post mortem interval (hours)	9.5	12
Fixation duration	4 weeks	1.5 years

Table S2. Parameters for segmentation steps.

	Workflow steps	0.94 µm voxel size		4.94 µm voxel size	1.88 µm voxel size *
Preprocessing	Spherical kernel for median filter	3 pixel radius	9 pixel radius	N.A	3 pixel radius
Edge extraction	Alpha value for Deriche-Canny algorithm	1	1	1	0.5
	Hysteresis thresholding (high / low threshold)	7 / 5	16 / 15	15 / 14	110 / 95
Post-processing	Irrelevant boundary removal	N.A.		Tissue-paraffin boundary was masked out	Edges introduced by circular field-of-view was masked out
	Minimum volume	3000 voxels		2000 voxels	5000 voxels
	Spherical kernel for closing operation**	5 pixel radius		10 pixel radius	11 pixel radius
	Two dimensional fill holes operation	Applied to XY, YZ, XZ planes			

* The dataset with a 1.88 µm voxel size is obtained by downsampling of the 0.94 µm voxel size image.

** For 0.94 µm voxel size data, we used ‘Morphological Filters (3D)’ function from MorphlibJ plugin. For 4.94 µm and 1.88 µm voxel size data, we used ‘3D Binary Close Labels’ function from 3D ImageJ Suite plugin on labeled image to avoid creating false connectivity that might occur with the large kernel size.

Blood vessel remains in vascular space

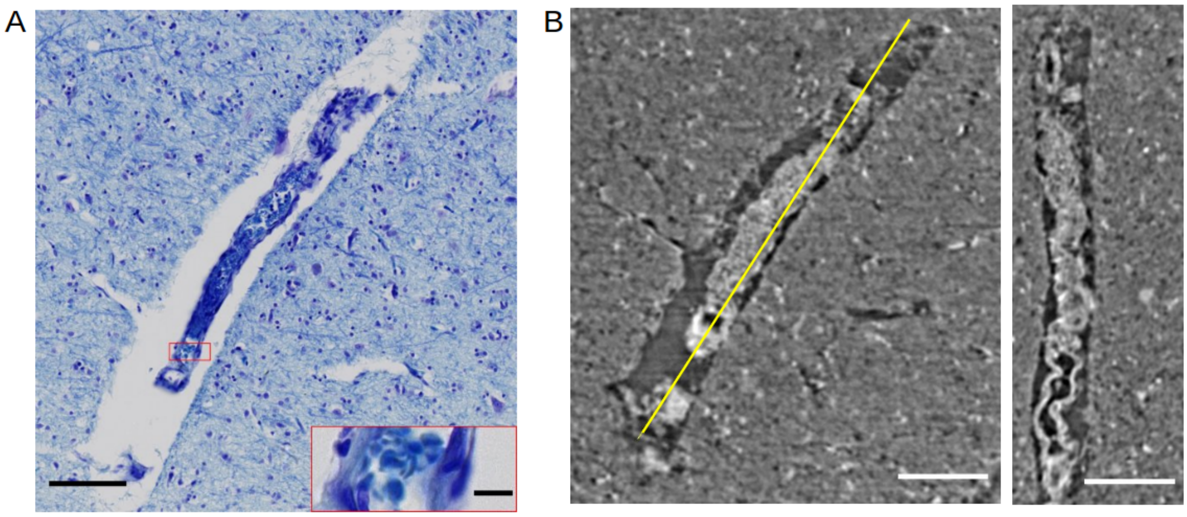

Figure S1. Red blood cells and vessel walls. **A**. Microscope image of a Klüver-Barrera stained section including blood vessels. The zoomed inset image shows red blood cells stained in blue color within the vessel walls stained purple. **B**. Left panel shows the phase-contrast microtomography matching the region of image A from the same specimen. Right panel is the phase-contrast microtomography sliced through the yellow vector displayed in the left panel, showing the endothelial cells of the vessel wall that appear to have detached from the tissue. Scale bars = 100 μm. Scale bar from inset of A = 10 μm.

Segmentation validation

To validate the segmentation method used in this study, we used the vasculature from 0.94 μm voxel size image and randomly selected 100 voxels within the segmented vasculature from each region of interests. Then, we visually determined whether they were truly vessels (Fig. S2A, S2B) or not (Fig. S2C, S2D) by observations of the phase-contrast microtomography. Some voxels were easy to determine (Fig. S2A). For less obvious voxels, we checked whether or not a vessel could be observed in the adjacent slides (Fig. S2B-D).

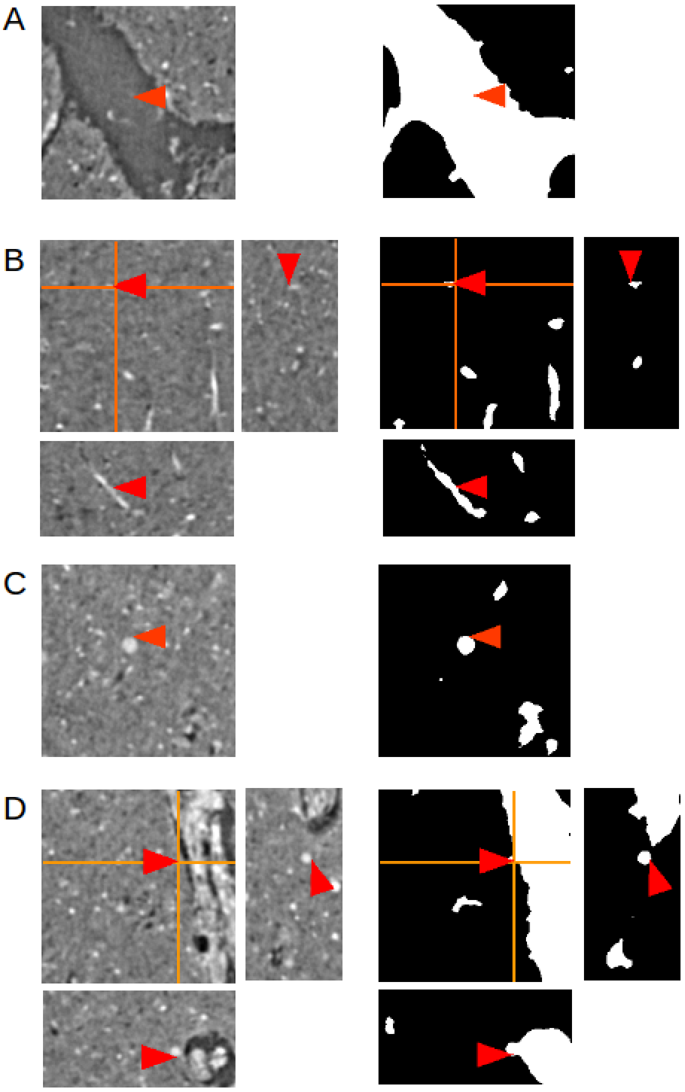

Figure S2. Validating the segmentation. Left panels show the phase-contrast microtomography. Right panels show the binary image where the segmented result is shown in white. Arrows point to the randomly selected voxels within the binary vasculature. **A-B**. Examples of true positive voxels. **C-D**. Examples of false positive voxels. Orthogonal sections are presented in B and D to better demonstrate the position of the selected voxel. All images have width and height of 100 μm and the orthogonal planes of B and D have the depth of 50 μm.

Qualitative validation

We compared the result of the segmentation from phase-contrast microtomography with CD34 stained microscopy data. Because of the presence of strong auto-fluorescence, this validation was considered qualitative. Many vessels identified by phase-contrast microtomography also showed strong CD34 staining.

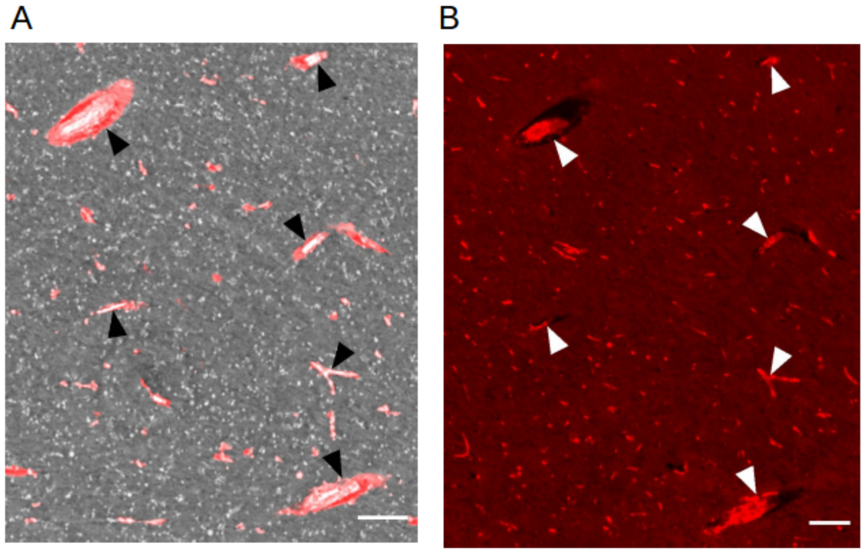

Figure S3. Vessel segmentation from phase-contrast microtomography compared with CD34 stain. **A**. Maximum intensity projection of 0.94 µm voxel size phase-contrast microtomography over 10 µm depth overlayed with segmentation result in red. **B**. Approximately matching region of a CD34 stained section. The arrowheads indicate vessel structures that are shown in both phase-contrast microtomography of unstained tissue and CD34 stained tissue. Human brain is known to accumulate auto-fluorescence throughout aging, so not all bright regions can be referred to as blood vessel. Scale bar = 100 µm.

Region of interests from phase-contrast microtomography

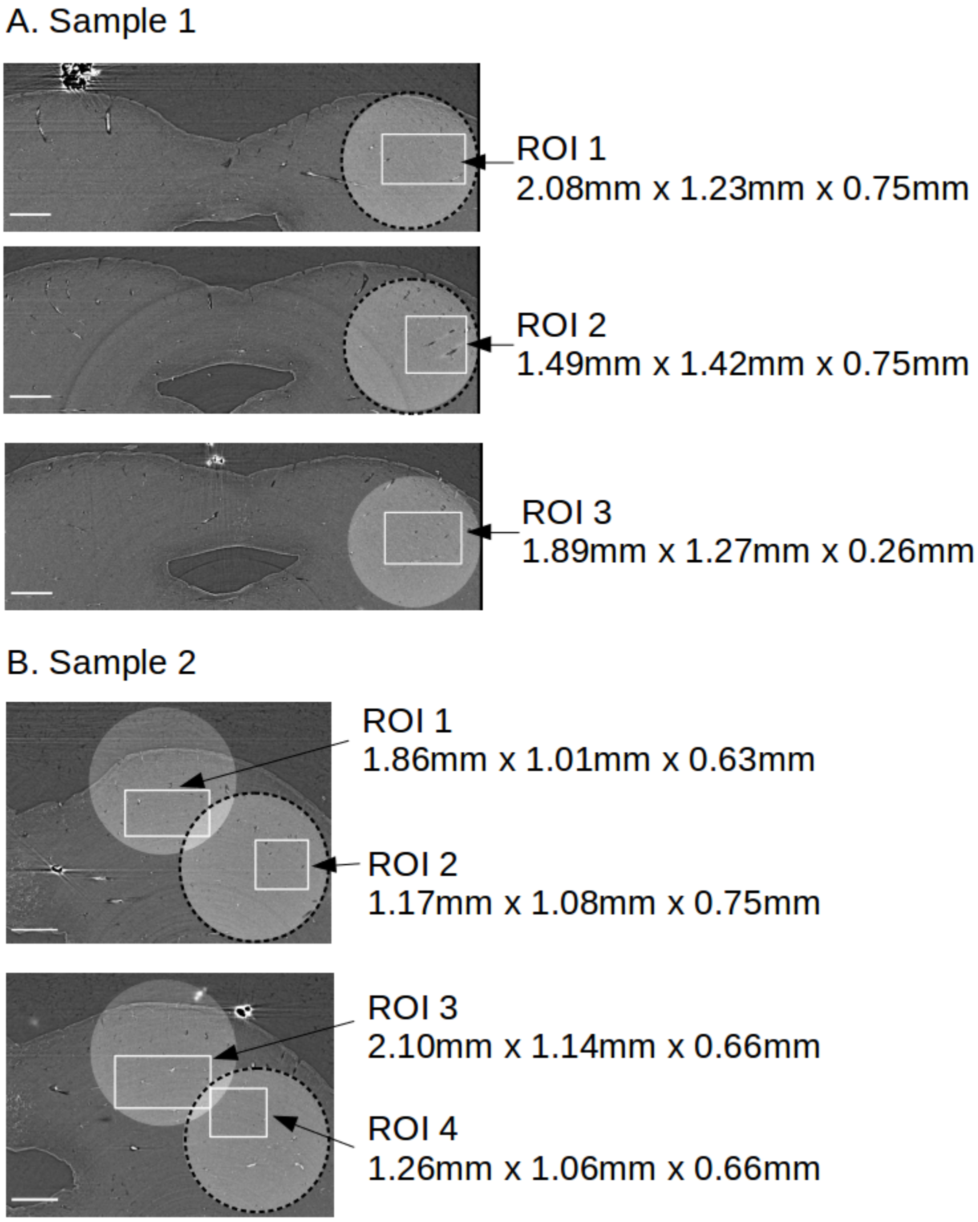

Figure S4. Summary of microtomography dataset. **A**. Region of interests (ROIs) from sample 1. ROI from 4.94 μm voxel size image is overlayed with field-of-views of 0.94 μm voxel size images. 0.94 μm voxel size images were acquired in three different Z positions. The rectangles indicate the ROIs selected for 0.94 μm voxel size image analysis. ROIs were selected where artefacts such as air bubbles or ringing artefact were not present. The width, height and depth of the ROI from 4.94 μm voxel size image is: 11.50 mm, 4.07 mm, 1.55 mm respectively. **B**. ROIs from 4.94 μm voxel size image of sample 2 is overlayed with field-of-views of 0.94 μm voxel size image. Superior colliculus of sample 2 was larger than sample 1 in medial-lateral axis and smaller in rostral-caudal axis. Thus, 4.94 μm voxel size image covered only one hemisphere and 0.94 μm voxel size image was acquired in two different XY positions. Each XY positions were scanned at two different Z positions. The width, height and depth of the ROI from 4.94 μm voxel size image is: 7.02 mm, 5.22 mm, 1.38 mm respectively. (**A, B**) Field-of-views circled with dashed lines were downsampled with 0.5 ratio for vessel length estimate analysis presented in section “Length estimates of short and long peripheral collicular vessels”. Scale bar = 1 mm.

Tissue shrinkage factor

In order to calculate the tissue shrinkage, we scanned sample 2 before and after paraffin embedding using different imaging methods. Prior to paraffinization, sample 2 was scanned using 14.1T MRI (Bruker Biospec, Ettlingen, Germany) using gradient echo (repetition time = 30 ms, echo time = 3 ms) with 200 µm voxel size. After paraffin embedding, the sample was scanned using cone beam microtomography (Tomolab station at Elettra, Trieste, Italy) with 20 µm voxel size. The sample volume was calculated using ITK-SNAP segmentation tool. The volume reduced to 50 % after paraffinization. We assumed that the shrinkage in X, Y, Z directions were equal leading to : Shrinkage factor = $\sqrt[3]{0.5} = 0.8$

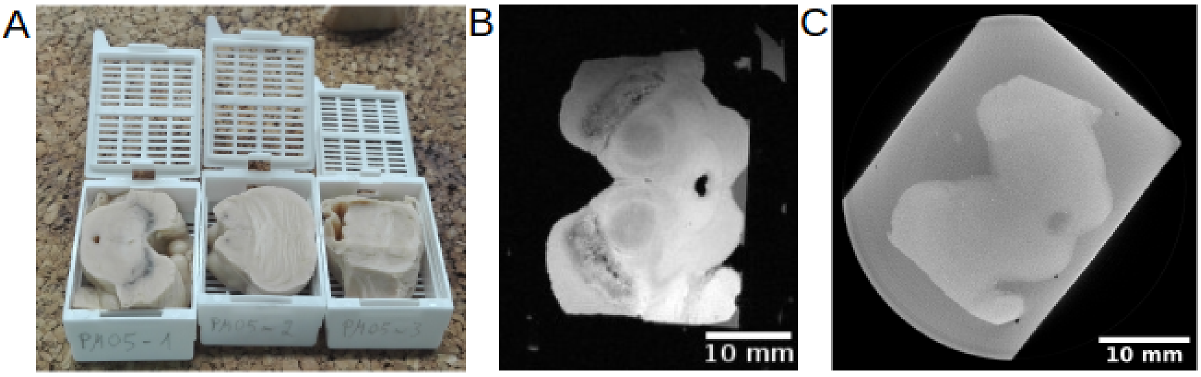

Figure S5. Tissue shrinkage before and after paraffinization. **A.** Photograph of sample 2 after fixation. The left section which includes superior colliculi was used in this study. **B.** Structural MRI image of sample 2 before paraffinization. **C**. Cone beam microtomography of sample 2 after paraffinization.

Video

Phase-contrast microtomography (0.94 μm voxel size) overlayed with vessel map in red. Region of interest (ROI) shown here is ROI3 of sample 2 (See Fig. S4).
